# Well-being dialogue: Elderly women's subjective sense of well-being from their course of life perspective

**DOI:** 10.3402/qhw.v7i0.19207

**Published:** 2012-12-10

**Authors:** Ann-Marie Svensson, Lena B. Mårtensson, Ulla H. Hellström Muhli

**Affiliations:** 1 School of Health Sciences, Jönköping University, Jönköping, Sweden; 2 School of Life Science, University of Skövde, Skövde, Sweden; 3 College of Nursing, University of Rhode Island, USA; 4 Department of Sociology, Uppsala University, Sweden

**Keywords:** Well-being, elderly women, narrative, lifeworld, case study, qualitative research

## Abstract

In this article, we are concerned with narratives of elderly women's well-being from their perspectives of the latter parts of their life, living at special housing accommodation (SHA) in the context of Swedish elderly care. In focusing on narratives about well-being, we have a two-fold focus: (1) how the elderly women create their own *identity* and *meaning-making* based on lifetime experience; and (2) how narratives of well-being are reflected through the filter of life *in situ* at the SHA.

Based on empirical data consisting of well-being narratives, a dialogical performance analysis was undertaken. The results show how relationships with important persons during various stages of life, and being together and enjoying fellowship with other people as well as enjoying freedom and self-determination, are central aspects of well-being. The conclusions drawn are that the characteristic phenomena of well-being (the what) in the narratives are continuity, identity, and sociality for the elderly person, and this is manifested (the how) as a question of contrasting the state of self-management and self-decline.

With reference to The National Board of Health and Welfare (2011), the population of old people (out of which 70% are women) living in special housing accommodation (SHA) for elderly people in Sweden are satisfied with the safety, treatment, health care achievements, and the living environment within the SHA. However, the elderly report having limited opportunities to influence the amount of time in getting help. Furthermore, they express dissatisfaction with how the care professionals consider their points of view and wishes. The elderly persons also view the social interaction and community at the SHA as less positive, in addition to having fewer possibilities to do different kinds of things that they enjoy, for example, being able to go outside when they want to. Such reports indicate lower well-being and are well known from previous literature (Berglund, 2007; Harnett, 2010; Hellberg, Augustsson, & Hellström Muhli, 2011; Jacelon, 1995; Porter & Clinton, 1992; Svensson, Bergh, & Jacobsson, 2011). Other indicators of lower well-being are, for example, higher consumption of tranquilizers among females (74–84 years old) than among males of the same age (Tornstam, 2007). This raises a social concern regarding the lack of an individually-suited environment for the well-being of elderly persons living at SHAs. With this in mind, we are concerned with the individual perspectives on well-being, and more specifically, on the dialogic/performance specific nature of narratives given by elderly women, when making sense of the subjective well-being from the perspective of their later life living at SHAs. In focusing on narratives about well-being, we have a two-fold focus: (1) how the elderly women create their own *identity* and *meaning-making* based on lifetime experience, and (2) how narratives of well-being are reflected through the filter of life *in situ* at the SHA.

## The narrative

The term *narrative* holds many meanings and is used in a variety of ways by different disciplines. It is often held synonymously with *story* (Kohler Riessman & Quinney, 2005). Here, according to Kohler Riessmann and Quitnney (2005), we give the term “narrative” a definition as *long sections of talk—extended accounts of lives in context over the course of single or multiple interviews*. The act of telling can serve many purposes, such as remembering, arguing, justifying, etc. An elderly person who provides a story about the conditions of well-being at the SHA gives a narrative that is meaningful to her, and at the same time it describes her experiences and feelings about her everyday living condition. The well-being narrative—a form of case study—embodies the experience of the individual and her *lifeworld* (Husserl, 1930/2004; Merleau-Ponty, 1962; Mishler, 1984; Riessman, 2002), or the way that meaning of well-being is perceived (Heidegger, 1927/2004). In this study, we draw attention to how the elderly women narrate about themselves, their feelings and about occurrences from the past as well as from the present, as a human interaction in relationship with the author.

## Well-being

The concept subjective well-being has attracted attention from diverse academic disciplines; but it is quite vaguely defined and described. There is also a number of critical arguments, that the concept of subjective well-being is associated with contestable, morally-laden ideas about happiness and the good life (Hanlon & Carliste, 2008). Furthermore, well-being has highly individualistic connotations and is uncritically associated with consumer culture of Western economics (ibid). According to Sarvimäki (2006), from a caring science perspective, well-being entails both health and quality of life, whilst “being well” is one aspect of health, which refers to the phenomenological concept of emotions and experiencing. Concerning elderly women's experiences of well-being as a constituent of the meaning of life, their own homes, families, and neighbors are primary important dimensions; thereafter, follows psychological and social well-being and health (*ibid*.). According to Sarvimäki, the quality of life is about well-being and two other criteria account for what a good life is. The first criterion is a *hedonistic* criterion, from which the life can be valued, namely from the degree of lust, reluctance, pain, and painlessness. That means a good life consists of feelings of pleasure, connotations of happiness and satisfaction, and in a longer time perspective. The second is that a definition of quality of life as a whole can be described as a complicated concept, usually referring to the central dimension of quality of life and the possible causes and relations between them as subjective experiences of meaning, and to the objective aspects such as somatic health, professional situation, accommodation, and the family situation. As appears, the quality of life and well-being are two separate concepts even if they are related to each other.

Health was defined by the World Health Organization (WHO) (1946) as follows: *Health is a state of complete physical, mental, and social well-being and not merely the absence of disease or infirmity*. Therefore, health is about well-being within several dimensions, including the physical, mental, and social aspects. Dahlberg and Segesten (2009) state that the essence of health is the experience of well-being—which means to feel good and that one has a good everyday life, but well-being means more than that. In order to experience health and well-being, there must be an experience of *being able to*, and the essence of health is that a person is *able to fulfill her/his minor as well as major goals* in (everyday) life, for example, *is able to carry through minor and major life projects*, (Dahlberg & Segesten, 2009, pp. 101–102). Given the meaning of health and well-being described above, care professionals must then somehow, touch the client's/patient's world, for example, the lifeworld that also includes the existential view. Thus, the care has to be lifeworld-led care (ibid.). Wånell (2008) describes elderly persons’ well-being in similar words but reference is also made to the group level of elderly′s well-being and to society. Therefore, fundamental to the well-being of the elderly is the ability to act and to realize vital goals. However, lack of health can hinder the freedom of choice to form one's life. The interface between society and the individual is therefore of importance (ibid.). As a result, caring interventions should be on different levels, on the individual, the group and on the societal level.

On the individual level of supporting well-being at a SHA, the caring relationship is the space in which caring takes place and in this caring relationship a healthcare relationship is created. Caring is about initiating the elderly person's health and well-being processes by providing room for growth and development (Mayeroff, 1999; Wiklund, 2003).

Therefore, questions that can be asked in this context include: how does the elderly woman experience well-being in a SHA from the perspective of lifetime experience? How are the well-being conditions described at SHAs? To generate scientific knowledge on the topic, the aim of this study was to understand and describe elderly women's well-being from their perspectives of the later parts of their life, living at SHA. The underlying data collection is comprised of narrative interviews with elderly women living at SHAs. This will be presented in the results as one case, in terms of a fictitious woman named Ella.

## Methods

### Design

The design of this study is characterized by dialectics between a theoretical guiding and an inductive interpretation and the analysis of the narratives about well-being are related to the key concepts *identity, meaning-making*, and *lifeworld*. The design has its starting point in the theoretical approach which claims a sensitive attitude to the human, by using a narrative methodology (Kvale, 1997; Ricoeur, 1976). Ricoeur (1983) holds that narrative can be that which solves tomorrow's *aporia*, with the purpose of arranging separate events into a meaningful wholeness. This theoretical approach enables an understanding of an activity that not only is an articulation of being-in-the world, but also constitutive for the meaning of being-in-the world (Heidegger, 1959/2012). These kinds of philosophical implications mean that realities are constituted through language and are organized and maintained through discourse and in narratives (Linell, 2012). This approach is concerned with the intersection of individual experiences and of realities with those of larger context and, in this study, as it relates to the experiences of well-being at a SHA. This is the living process of well-being, which is sculpted in the narratives through a connection with the life course (Ricoeur, 1983). In this study, it aims to highlight the elderly women's lifeworld of well-being from their own course of life perspectives. This implies, on the one hand, a dynamic interpretation of what personal narratives are about, and the structures of meaning in the narratives about well-being and identity while living in a SHA, on the other.

### Settings

This study was conducted between April and June 2011, at three different SHA facilities within two municipalities in western Sweden. Five elderly female residents were the participants. In accordance with the Helsinki declaration of 1975 as revised in 2008 (WMA, 2008) and with the Swedish Government (SFS, 2003:460), ethical aspects have been discussed. The study was approved by the Regional Ethical Review Board, University of Gothenburg, Sweden, Reference number. 660-10. The informants were given both written and oral information about the four ethical principles in research, namely, autonomy, beneficence, non-malfeasance, and justice—as regulated in the Declaration of Helsinki (WMA, 2008). The participants were informed about the study as well as the issue of privacy, that their identities would not be revealed and that participation was voluntary. All five participants agreed to participate before the data gathering (interviews) prior to the actual meeting and to have any possible questions answered. Ethical authorization was obtained from the senior managers of the SHA. Both oral and written consent was obtained at the time of the interviews.

### Participants

The elderly participants were aged 75–100 years (all female). All participants were cognitively well-functioning and could carry on a conversation. They had lived at an SHA from between two and seven years.

### Procedure

An information letter about the study was sent to the head of SHAs, who also informed the elderly residents about the study. Those who were interested in participating received both written and oral information about the study. The head of SHAs also suggested informants should meet the set of inclusion criteria related to the individual's health-records. Inclusion criteria included: having lived in an SHA for at least six months, being able to speak the Swedish language, and being able to hold a conversation. Individuals with dementia disabilities and with impaired decision-making skills were excluded. All interviews took place at the informants’ homes at the SHA.

### Data collection

The first author conducted the interviews, which varied between 25 and 50 min. The interviews were recorded on an MP3 player and began with open-ended questions such as: (1) Can you tell me about your life, who is Ella? (2) How do you experience the everyday procedures at the SHA in order to feel well-being? The interviews were conducted as dialogues in which the individual elderly person could tell their story as a dynamic co-production between the teller and the listener. During the interviews, follow-up questions such as “How did you experience that?” and “What was well-being for you then?” were asked in order to gain an in-depth understanding of their narration and to understand what well-being meant for the specific elderly woman. The interviews were transcribed verbatim (Linell, 1994, 2004; Riessman, 2008). In all, the interviews generated 74 pages of transcribed text with a spacing of one and a half lines.

### Data analysis

Narrative analysis is a method in which people's perception of reality is made visible, namely, how that person experiences their lifeworld and the context in which events are played out (Riessman, 2008). In this study, there is also an analytical perspective on the dynamic aspects of narratives. That means a close dialogic/performance analysis by asking who, when, and why that is, for what purposes? (Riessman, 2008). Accordingly, analytic attention is given to how the facts got assembled *that* way in the story? Furthermore, the narratives have been analyzed, as well as the storyteller, and how the story is told related to the context (Ricoeur, 1983). The approach for the analysis was to view all five narratives as one case for the purpose of identifying the thematic content structure in the narratives (McCance, McKenna, & Boore, 2001; Polkinghorne, 1988).The analysis has been performed in following steps: First, we (author one and three) listened to the five interviews several times in order to acquire a full appreciation of the data. Second, the interviews were transcribed (by author one) as a talk interaction between the interviewer and the interviewee) (e.g., Interviewee Ella = E: *we had to watch every penny*. Interviewer = I: yes:::,. E: there wasn't the extravagance like there is today. I: no). These transcriptions were read and re-read (by all authors) to elucidate several themes, and an analyzing process took place in the form of coding, in which the text fragment was placed in topic clusters. These clusters were analyzed in order to find specific content of what (what is spoken) and how, who, when, and why (dialogic/performance) in the narratives. Thirdly, the themes and course of events were sorted in chronological order in the narratives (by author one). Forth, the chronological themes in the narratives were closely read through once again (by all authors) to ensure the various elements of the meaning of well-being through the life course and how these meanings were accounted for. Fifth, three themes were selected (by all authors) for the final analysis: (1) childhood memories as a source of well-being, (2) family and work a source of well-being, and (3) well-being opportunities at the SHA. This analysis is presented below for transparency and it shows the application of theory to the analysis by including the textual material (the data) from the narratives. (Nixon & Power, 2007; Winter Jørgensen & Phillips, 2011).

## Results

The following text presents events that the elderly living in SHAs describe as experiences that are important for their well-being. Important for well-being are fellowship and social interaction, and being able to enjoy a sense of freedom and independence. The narrations of well-being begin with a story taking place in the person's childhood, continuing into adult life, and concluding with a contrast of how life is now in the SHA. Consequently, well-being in the present is reflected through a filter of the life one remembers, and the elderly choose and evaluate different events and experiences occurring during the course of their lives. This comprises the main structure of the narratives. In the following results, this chronology is shown (1) partly through what the idea of well-being is for the elderly, and (2) partly how well-being is described. The dialogues are shown as a product of the interaction between the listener (the interviewer) and the teller (the elderly woman).

### Transcript notations

The following transcription conventions were used: (.) signals untimed micro-pause; (2 s.) signals micro-pause in seconds; [] in square brackets indicates positioning; £ indicates laughter in the speaker's voice while saying the enclosed word; underline indicates the increased emphasis; :: extended words; (mm) characters in parentheses are the interviewer's.

In all the transcripts that follow, I = Interviewer, E = Ella, a case-constructed person.

### Childhood memories as a source of well-being

When the elderly Ella recounts what brought her happiness and well-being in life, thoughts go back to events in the past, when relationships with others were the focal point. A clear pattern in these childhood relationships appear as essential portrayals in the narrations of well-being. In the following extract, Ella tells of a childhood where she, as a child, experienced feelings of both freedom and well-being, despite the adverse economic situation of her family.

Extract 1. A narration of what creates comfort

1. E: yes we were children (.) we had fun (.) we got to be free y'know (2 s.) get rid of all these (2 s.)
2. must must must ( mm) like it is today (2 s.) but of course (.) we didn't have (2 s.)
3. we didn't have money in those days to be sure, like it is now.
4. I: no:
5. E: we had to watch every penny.
6. I: yes::
7. E: there wasn't the extravagance like there is today (2.)
8. I: no::
9. E: all of us (mm) all of us had such a nice time together

As seen in the above extract, this dialogue includes three analytical levels: (1) the text for the concrete account that the listener or reader can take note of; (2) the actual story or core (experience of well-being); and (3) the story as it was told (the *fabulan* or order of events the teller recounts, for example, in the order or time frame) (Hydén & Hydén, 1997).

The story of well-being as told by the elderly woman starts with when she was a child and they had fun. The “we” in line 1 refers to the children she played with; thus, the elderly woman uses the plural form encompassing all the playing children. Not only did they have fun together through play, they were also free, “we got to be free y'know” (line 1). That little expression “y'know” shows two levels: (1) It acts as the causal connection between play and freedom; and (2) it shows interaction between the teller and the listener. This connection is a so called *in situ* construction based on a retrospective adult perspective. In the narrations, the temporal dimension of time is made visible. The elderly woman places the enjoyable experiences of childhood at a point in time (Hydén & Hydén, 1997), and the content is clear in comparison to how she as a child could have fun despite the poor economic situation, “we didn't have money in those days to be sure, like it is today” (line 3). She compares this to the children of today, “there wasn't the extravagance like there is today” (line 7). The narration concludes with (*the plot*) the narrators point, that is, that despite this, “all of us had so much fun” (line 9), namely, that money and abundance is not needed to create comfort, but rather it is about being free and without obligations and the must-haves (line 2).

The story also highlights what Tornstam (2011) means by the inner core of social networks, that is, the relationship to friends (and family). Innermost relations such as the affinity with others are identity supportive social resources, and promote the creation of identity and meaning in the elderly person's situation, not only during childhood but also in their current situation at the SHA.

What is interesting here is how the elderly woman allows the listener (the interviewer) to understand the event as a dynamic description of well-being by contrasting her childhood moments of joy with the present. To be kids, to have fun, and as a child to be carefree and without worries depicts well-being, in contrast to the present where everything is about musts (line 2).

As seen here, the extract concerns not only meaning-making between the elderly woman's past and the present situation at the SHA in relation to well-being, but also the more active dialogical understanding in the account between the listener and the teller. From this account, the situation (*in situ* negotiations) also affects the subject's identity. It is, therefore, not only an account of a carefree time long ago, but also an example of how the elderly person retains her identity through the recounting of earlier events for the listener.

The feeling of fellowship experienced in childhood is recounted with emotion, including the joy that was felt when being with family during family activities such as playing an instrument and singing together, both within the family unit and also at parties. The well-being described in the following extract is that of being a part of a social situation. Ella and her father are the central characters.

Extract 2. A narration about a musical father, a good time, and a nice family home as a basis for well-being

1. E: oh::father was (Ella straightens up) so musical it didn't matter what instrument you gave him.
2. he could play it (2 s.) so:: there was always song and music at home (1 s.)
3. I: mm
4. E: when they would go out for a ride (.) they have told me (.) some booking
5. where they would play (.) so don't forget that Ella is coming too this they have
6. told (hm) me (.) so I could go with them they thought is fun that I was there and played too
7. I: how did you experience that time?
8. E: well:: (.) yes I thought that it was a rather good time
9. I: I see::
10. E: I have always felt happy and we had such a nice parental home
11. I: yes
12. E: there was singing and music every day and good food

It is interesting how the home is portrayed as an ideal place (lines 2, 12). The woman tells of her own subjective account that she was proud of her father's musicality, by straightening her posture (line 1) when telling her story. In this manner, she creates a certain dynamic and excitement in the narrative (Hydén & Hydén, 1997), while showing her affiliation to her musical father, and her own musicality.

Extract 2 contains different aspects of well-being, and their origins in childhood. The elderly woman describes that she was needed and sought after (line 6), which supports her current sense of self-worth (Heap, 1995). Through the dialogue, the elderly woman explains that she is desired and needed. She shows what she thinks of her own musicality, and even how others saw her. Accordingly, an identity creation or construction has taken place, through music and within social interaction with others, and she maintains who she was and who she continues to be. According to Mead and Morris (1967), this model of identity creation occurs during an interaction between how an individual sees himself or herself and how others see him or her. This interpretation of the story reinforces the self-identity of the elderly woman and a sense of self-worth by describing not only the father who was musical and in great demand, but also she herself (line 6). This form of identity creation is based on the retrospective force on life and presents a picture of how and where the identity of the elderly woman has been created, and by recounting an episode from that time, maintains that identity (Eriksson & Eriksson, 1998; Tornstam, 2011).

### Family and work a source of well-being

Even if childhood is essential in creating a feeling of well-being in the present time, Ella describes the period when life was all about building a family and working, as a life filled with joy and happiness. Having a family forms a relationship in life, and comprises a unit where everybody lends a hand with everyday work and tasks. Although life was hard, there was pleasure to be found. In fact, it appears that joint efforts and work was a factor that generated happiness and well-being, as shown in extract 3, below.

Extract 3. A narration of sticking together and work as a source of joy and well-being

1. I: how was your life when you had the farm in x-town (.) and the children were at home?
2. E: it was full to the brim (.) and there was work (.) and everyone was happy
3. I: what was it in life that made you happy?
4. E: well:: I was happy for the work and it all went well
5. I: mm
6. E: and:: we helped each other with joy
7. I: yes
8. E: as long as one could manage

In this account there are primarily two levels of analysis: (1) How the woman creates two dynamic forces that form the subjective experience of well-being, namely, the joy of positive development and direction in life, “it all went well” (line 4), while simultaneously being characterized by a laborious life, “it was full to the brim (.) and there was work” (line 2).

Analysis level (2) is about the basic core of the story of adult life, namely, that they helped each other in the family (line 6), which suggests the desire for intimacy and a relationship with the family members, a kind of fusion of self to others in an emotional and work related manner. Eriksson and Eriksson (1998) describes this phase of life, *early adulthood*, as a psychosocial phase moving from the creation of identity in childhood to being prepared to engage in an intimate relationship with a partner. Eriksson and Eriksson (1998) uses the concept of *generativity* to describe human strength or self-qualities that arise during this phase of early adulthood, and it is expressed through caring for children, family, and loyalty to the family despite the situation. This is described in the elderly woman's account as an involvement in the care of the family: In the elderly woman's narration, this analytical model of her identity as commitment to the care of the family is expressed in “it was full to the brim (.) and “there was work” (line 2) at the same time they were happy for the work and it went well (line 4). Thus, the commitment during this period in life was to build up sustenance for future generations and to bring up their children.

Ella continues her account and has reached the period in her life when the children have grown, and she describes a historical presentation of thoughts to start work outside the home environment. She tells of being contacted and asked to start work as a switchboard operator. She had worked as a switchboard operator for a brief time earlier in life, and this would become important at this stage.

Extract 4. A narration of how she refused a job offer

E: then I was at home with my children (.) and::: I thought I should be home for a while
(hmm) so I was at home with them
I: I see::
E: a::nd then when I had been at home, they had opened a hospital (1 s.)
they built (.) opened (.) in 73
I: I see:
E: and then they phoned me £an old switchboard operator £ (.)
I: oh, I see
E: but I didn't want to start working then because I wanted to be at home
with the children (1 s.) so I didn't want to start then (.) so I started (.) 1976 (.)
so I started at the hospital's switchboard there
I: okay
E: then oh::: it was very nice
I: oh::: what memories you have of the years
E: so there I was until retirement (.) 94
I: oh my
E: yes::: so it became my life

In the above excerpt, it is interesting how the woman's narration contains information that goes beyond the story, that is, a dynamic and exciting narrative strategy of how she rejected a job offer although she was called and offered a job (lines 6, 8). She was hesitant at first of the offer (line 7) to work at a newly built hospital that opened in 1973, work that should have been attractive for many at that time. Instead, she preferred to be at home with her children for a few more years. Only three years later did she accept the job offer, (line 9). The woman positions herself beside the events of the account and does what Labov (1972) calls external evaluation of decision through her comment, “I wanted to be at home with the children.” This shows that she evaluates her decision as justified. The decision to start work after several years, and that the offer was still open verifies the correctness of the decision. The woman recalls this period as a nice time (line 12) and this shows that it gave a positive impression that she still retains.

In just a few sentences, Ella shifts from the period when she was at home with her children (line 1) and her work as a switchboard operator (line 10), to when she retired (line 14) and concludes in the present with: “yes so it became my life.” Through these words, she links meaningful events in her life that have created joy and well-being, and her own role in the events. With drama and relationships with other people, the account concludes with the statement, “so it became my life.”

### Well-being opportunities at the SHA

Ella's accounts of events move through the years, and we shall discuss what brings her joy and a sense of well-being in her life at the SHA. In the extract below, the woman describes frequent visits, but despite this there is a longing for those who are close to her. The relationship with family members, and especially the children, is very important for the woman. Although family is the closest to her, keeping in touch with friends is also important for her well-being. The following extract describes the importance of both these relationships.

Extract 5. A narration of children's visits and friendship's significance as a source of well-being

1. I: if there is something today that makes you feel good (.) that is a source of well-being (.)
2. what would that be?
3. E: well:: it is all my friends and my children
4. I: I see::
5. E: especially my children (1 s.)
6. I: I see::
7. E: I miss them so terribly sometimes so (1 s.)
8. I: mm
9. E: and yet they visit so often
10. I: mm
11. E: but it is just as sad every time they leave
12. I: yes:: have you told them how much they mean to you
13. E: oh yes:: many times, every time (2 s.) they come

This account also displays knowledge of the inner core of social networks (Tornstam, 2011), that is, family relationships. Ella explains that her children specifically give her happiness (lines 3, 5), that is, those who are closest to the elderly woman, and who characterize the inner core of the network. She also expresses her longing for her children (line 7), despite frequent visits to the SHA by them. This longing gives expression for what Tornstam (2011) describes as the interrelations’ qualitatively most important quality, that it is the degree of depth or presence that characterizes the relationship. This cannot be experienced in the SHA environment, or with the people there, but requires contact with the closest relations. The story continues in extract 7, explaining how friendships created in the work environment continue to play an important role.

Extract 6. A narration about the impact of friendships for well-being

1. E: that I am as I am anyway
2. I: yes:
3. E: I have my friends (.)
4. I: yes::
5. E: still have my old friends you know
6. I: okay:
7. E: (2 s.) they come and:: visit me those who have worked at telecom (.)
8. when I worked in the fifties (.) a::nd (.) those at the hospital (.)
9. there are many (2 s.) I have many friends
10. I: mm (.) do you talk about both the old and new things then
11. E: yes:: we talk about both
12. I: is it important to have these contacts?
13. E: very important (.)
14. I: to have those contacts that you have had for a long time
15. E: yes:: it's very important (.) that they don't forget me
16. I: maybe they see you as a different person than the one you are today
17. E: yes:: they remember how I was when I was healthy
18. I: mm
19. E: but I don't know (1 s.) they like me for who I am now too
20. I: yes:
21. E: otherwise they wouldn't be coming

Ella begins by stating, “I am as I am anyway” (line 1). The function of her opening account is to begin the dialogue with a summary (Labov, 1972) and expresses her understanding of the continuity of her identity despite her age, environment, and current conditions at the SHA. She continues then with a description of time, or the temporal dimension of the past compared with the present, and the importance of her friend's visits so that “they don't forget me” (line 15). Despite life events being placed on the timeline, before the account continues, she says that she talks about both current and past events (line 11). These are perceived as very important (line 15). According to Tornstam (2011), the consequence of the importance of the friendship relations and social integration relating to well-being is interwoven. The woman says that she has many friends (line 9) and that these contacts are important to her.

Friendship and conversations with friends confirms the woman's existence both as it was (line 11) and as it is now (line 12). All of this identity construction, confirmation of existence, and retention of own identity is noticeable (line 19).

It is interesting in this account how the woman positions herself (Harré & van Langenhove, 1999) as a social person with many friends (line 9), and uses this to show (lines 3, 5, 9) that she is a socially competent person who has maintained friendships throughout the years (about 50 years).

Extract 7. An account of the relation between care staff and the subject as a source of well-being

1. E: I have to praise the staff; they are very kind and helpful (.) I (.)
2. they ask how would you like it Ella and how can we help you (.) they say (.)
3. so they are very accommodating that you are kind and competent who can help and such::
4. I: do you get along with the staff?
5. E: yes:: very well
6. I: mm
7. E: we can joke and talk about everything
8. I: is that important?
9. E: it is important (.) it is very important (.)

Even the relationship with the staff at the SHA and the care relationship are described by Ella as a subjective experience of well-being. In this relationship, the staff give nursing and also care. The woman praises the staff and describes them as kind and helpful (line 1) and says the conversations between them touch on most things (line 8), the act which is considered important (line 10). The staff provides help, which Ella confirms by describing how she herself wants to be helped. The extract below expresses the care relationship between the Ella and the care staff at the SHA.

This account shows two analytical levels: (1) the importance of the care staffs’ relationship to the woman; and (2) how the care staff constructs well-being within the woman using the relationship as a care tool. As seen in this extract, the dialogue between the staff and Ella is an interaction that leads to the enabling of a caring relationship. This interaction is described by Ella as friendly, “we can joke and talk about everything” (line 7) and that the staff are “kind and helpful,” (line 1). It is also important to “get along” with the staff (line 9). The extract shows that the care staff considers the woman's participation and self-determination by asking, “how can we help you Ella” (line 2). In this manner, Ella is acknowledged as a person who can make decisions, has her own-will, and who wants to make her own decisions. This strengthens the woman's meaning-making and self-image. This type of relationship is described by Mayerhoff (1999) as a caring relationship that can increase the woman's sense of well-being. The care staff listens to and learn how the woman wants to be cared for. Mayerhoff (1999) states that this helps a person to grow and be the person they really are. This social interaction creates well-being.

In addition to visits by family members and friends, life at the SHA is characterized by trying to fill the days with “meaningful” activities, as opposed to earlier in life when family and work filled the days. Activities can also be a source of well-being, and at the SHA, activities are arranged nearly every day. Ella appreciates this and tries to participate as often as possible. Activities include music, newspaper-reading, bingo, quizzes, and gymnastics.

Extract 8. A narration of meaningful activities as a source of well-being

1. E: we a::nd (1 s.) have something called the house of birds
2. I: mm
3. E: and there we get (.) there is the day's recreation [day-activities at SHA]
4. there are those who work there who have (1 s.)
5. we have different programs (.) today it was to be (.) gymnastics (.)
6. I: I see::
7. E: we have sitting down gymnastics on Tuesdays (.)
8. and there is always something (.) someone who comes and entertains a::nd
9. I: do you appreciate having something to do (.) and look forward to
10. E: £ I:: think that £ (.) I participate in everything (Ella laughs)
11. because I think (.) that one should participate

Even this extract displays two analytical levels that we wish to point out: (1) how the activities and programs organized for the residents of the SHA comprise a central dimension in the account, which may be considered a source of well-being; and (2) the institutional order which links events and people is also evident. Ella talks of activities like “The House of Birds” (line 1) and gradually describes the activity for that day and then the fact that there are different activities and programs (lines 5, 7). These activities do not appear to be arranged by the residents themselves at SHA, but by somebody else at the SHA, “and there we get (.) there is the day recreation [day-activities at SHA]” (line 3), “there are those who work there who have” (line 4). Thus, it is the staff that organizes and offers a repertoire of activities that the elderly person can participate in. It is shown here that the staff is the active ones and the elderly are the passive consumers, even when referring to activities. It is worth considering in this account whether the elderly persons participate in the activities offered by the SHA out of their own accord or due to an unspoken requirement to participate (line 10, 11) in order to show their appreciation of the staff's efforts.

With regard to the question of the appreciation of activities (line 9), Ella laughed when she answered, “I participate in everything because I think (.) one should participate” (line 10). Participating in activities may also be explained by the theory of exchange or reciprocation, a supposition of people's give and take (Tornstam, 2011). For the elderly there is an imbalance in the reciprocity, when the elderly often are the recipients of different advantages. To “attend” could, thus, be explained as a way to balance this relationship.

However, it is not only meaningful activities which are a source of well-being at the SHA but also the hope or the dream of regaining their own home, to cope and be one's own master. This involves being independent and having the freedom to decide when events occur or are performed. In retrospection, the woman looks at the bygone time in her own home and describes it as wonderful (line 3 in the next extract).

Extract 9. A narration of well-being reflected in physical ability

1. E: yes, of course, there I had everything around me (.) that I myself (.)
2. E: there I had everything around me (.) then I could do what I wanted to (2 s.)
3. E: but, of course, I think about how wonderful things were when I was healthy (.)
4. E: a::nd being at home
5. I: what do you think was so good then
6. E: it was so nice (.) I was my own master a::nd
7. I: yes
8. E: and take care of myself a::nd (2 s.) could go, go to the toilet when I wanted to and::

This extract reflects the physical ability in the past as a source of a feeling of well-being, independence, and freedom. This feeling is in contrast to life as it is now at the SHA. Past and present create a touching moment during the interview. In the past, she could take care of herself (line 8) and be her own master (line 6). This compares to life at the SHA where taking care of one's basic needs, such as going to the toilet when one wants to, are not possible.

The woman repeats the word “go” (line 9) in “could go, go to the toilet when I wanted to,” which expresses something the woman wishes to emphasize. The meaning of repetitive words, according to Labov (1972), includes the internal evaluation the person makes of herself, and what in this case the woman views as important to the account, that is, the desire to go to the toilet when she wants to. Correspondingly, the time at home when she was healthy, “but, of course, I do think of how wonderful it was when I was healthy” (line 3), and when she was at home (line 4), contrasts with her present time at the SHA, when she is ill. Here, the woman reflects upon the physical ability as well-being in the past, an effective method by the narrator to create meaning and show how she wants the account to be interpreted.

## Discussion

In this study, we have discussed five different accounts that have in turn been presented as one case study of the elderly's well-being. The aim was to understand and describe elderly women's (living at SHA) experiences of well-being and to find structures to facilitate well-being in everyday living conditions. The analysis shows that well-being is experienced as having *relationships with other people*, and experiencing *continuity of self-identity*, whilst there also occurs a type of *experience of self-renewal* caused by society and by others. *Well-being is, therefore, something that not only exists, but is also created in social situations with relatives, family, and with the care personnel the elderly women interact with on a daily basis at the SHA*. In this manner, well-being is described in terms and with meanings that seem obvious and which agree with earlier studies which describe the substance of well-being (Sarvimaki, 2006; Svensson & Hallberg, 2011). However, reminiscence is one of the substantial factors of well-being. This is performed through recollection and memory (Heap, 1995). The interesting issue about the contribution of this study is that that which creates well-being for the elderly person, must be found in past experiences in life as well as *in situ*. This experience creates the meaningful wholeness of arranging separate events and is what Ricoeur (1983) refers to as meaningful wholeness. The narratives *in situ* create an experience of renewal through dialogue about what the women experienced earlier in life through social contact. This knowledge is important for use in elderly care at the SHAs Because the SHA environment offers fewer confirmatory events or experiences for the elderly women, and these confirmations are important to strengthen the elderly women's well-being.

Life at the SHA is not only about being fragile and dependent on help, but also acknowledging that one's identity risks being erased. However, the women value events that also have meaning in the present, and the meeting place for this is the present moment when the woman talks *in situ* at the SHA. In presented extracts, Ella summarizes her life, and as shown earlier in the report, she says: “yes so it became my life.” This statement creates an experience of continuity in life at the SHA, and it should be highlighted. It expresses or defines the meaning that care should fill or offer, namely, to answer the statement “yes so it became my life,” with “so you continue to live.”

### Methodological considerations

In this study, we have chosen to use close-analysis to study in detail the women's narratives as one case. Accordingly, we have been able to examine the dialogical relationship between the researcher and the elderly women living at the SHA. Narratives are of a specific methodological importance to analyze experiences and identities in their multiple formations and in different contexts, here in the context of living at the SHA. This narrative methodological approach enables research on the changed meaning in life which influences a disproportionate number of women, for example, the fact that 70% of the old population living at SHAs in Sweden is female (The National Board of Health and Welfare, 2011). The individual narratives provide different perspectives on life when women face a lack of freedom in these kinds of factual situations.’ Bringing attention to individual narratives in interviews enables insight to the lifeworld perspective of the individual women at the SHA, with its own moral complexity.

The number of interviews that the resulting case is based upon may be considered to be few. Nevertheless, it is important to point out the difficulty in interviewing suitable informants in a study such as this as the elderly are often physically and mentally frail. Many women living in the SHA suffer ailments such as dementia and, therefore, have difficulty in holding conversations. This situation is also confirmed by Meinow, Parker, and Thorslund (2011), who noted in their research that fewer than 5% of those who lived in a SHA managed to conduct an interview and understand the information. Furthermore, it should be noted that the women who were interviewed at the SHA were of quite advanced ages, which made interviews lasting longer than 30 min tiresome for them. Such circumstances were handled with sensitivity and respect.

In the chosen method, context, analysis, and interpretation have been made transparent so that the reader can follow and understand how these relate to each other. In this study, we have shown how intimately connected and mutually dependent they are, despite the fact that they are often studied and described as separate subjects (Riessman, 2008). Narrating and listening, transcribing, and analyzing are interpretive tasks. The transcriptions have been reproduced so that the accounts are displayed as a result of an interaction between the interviewer and the interviewee. Another choice we made was for all reports from raw material to be built from selections of reality. This means that the specific extracts in the report are the researchers’ own choice. In this study the interviewer had a professional role (as researcher) in the conversation and had no earlier connection or relation to the elderly women in this study. However, the interviewers pre-understanding of the care context and of elderly persons comes being educated as a specialist nurse in the care of the elderly. This pre-understanding influences the interpretation and the understanding of the context for the narratives. This pre-understanding was a resource both to when information was given during the procedures for the study and to the interactions in the interviews.

The theoretical perspective chosen in this study allows for the creation of representations of reality where narrative creations *in situ* are presented, and the social practice between the researcher and the interviewee (the object of the study). This also applies to the interpretive interactional perspective, which is different from other qualitative studies.

## Conclusion

We began this study with the observation from previous research, which showed that many elderly people were partly satisfied with the caring in SHA. We also found, however, that there was lower well-being among elder persons at SHA, when it comes to for example self-determination. With reference to this study, we can now conclude that well-being throughout the process of narratives about events from childhood to the life *in situ* at the SHA is the *continuity* (of self-), *identity*, and *sociality*. These three concepts illustrate out the power of *continuous individual agency* that creates experiences of well-being. This power of continuous individual agency is the new knowledge gained in this study, and it has to be understood and explained as contextually-related. To conclude, this study contributes new knowledge about elderly women's well-being from the perspective of later life, living at SHA in the context of the Swedish elderly care. The new knowledge gained is: (1) that the elderly women construct their own identity *in situ*, based on their lifetime experiences of *relationships with other people*, and by experiencing *continuity of self-identity* in (2) narratives of well-being, reflected through the filter of life experiences *at* the SHA. The characteristic phenomena of well-being (the what) in the narratives are *continuity, identity*, and *sociality*. The “sociality” is displayed as knowledge of the inner core of social networks for the elderly women, and this is manifested (the how) *as a question of contrasting the state of self-management and self-decline*.

### Implications

The implication for care practice arising from this article is that care of the elderly at SHAs, and of elderly women specifically, is not only a question of delivering good physical care in home-like housing accommodation. Rather, it is a question of how the care is organized to support well-being at the SHA. The findings from this study highlight the importance of social relations and social interactions between the care staff and the elderly. They also highlight the importance of factors such as well-being and the maintenance of the social network. Accordingly, new methods could be developed for performing care which would promote well-being, for example, by caring interventions and by sanctioning from the care management level, to work in a lifeworld centered way. This kind of lifeworld centered care can promote the continuity of self-identity, as well as well-being of the elderly (women).

The utilization of care staff in the supporting approach of *continuity*, *identity* and *sociality* helps the elderly to re-author their identity and the personal reflected narratives of well-being, despite the physical and psychological declining capacity. This kind of care is lifeworld sensitive care and is performed by engaging the elderly women's world in a dialogue or interaction between the care staff and the elderly women. These findings are a contribution to operationalizing supportive care, and they represent an opportunity to improve the individual's well-being.

## References

[CIT0001] Berglund A.-L (2007). Satisfaction with caring and living conditions in nursing homes: Views of elderly persons, next of kin and staff members. International Journal of Nursing Practice.

[CIT0002] Dahlberg K, Segesten K (2009). Hälsa & vårdande i teori och praxis.

[CIT0003] Eriksson E. H, Eriksson J. M (1998). The life cycle completed.

[CIT0004] Hanlon P, Carliste S (2008). Thesis do we face a third revolution in human history? If so, how will public health respond?. Journal of Public Health.

[CIT0005] Harnett T (2010). The trivial matters. Everyday power in Swedish eldercare. (Dr. diss.).

[CIT0006] Harré R, van Langenhove L (1999). Positioning theory: Moral contexts of intentional action.

[CIT0007] Heap K (1995). Samtal med äldre. Om kommunikation, minnen, kriser och sorg.

[CIT0008] Heidegger M (1927/2004). Varat och tiden.

[CIT0009] Heidegger M (1959/2012). På väg mot språket. [Unterwegs zur Sprache] Translated by Sven-Olov Wallenstein and Ola Nilsson.

[CIT0010] Hellberg I, Augustsson V, Hellström Muhli U (2011). Seniors′ experiences of living in special housing accommodation. International Journal of Qualitative Studies on Health and Well-being.

[CIT0011] Husserl E (1930/2004). Idéer till en ren fenomenologi och fenomenologisk filosofi.

[CIT0012] Hydén L.-C, Hydén M (1997). Att studera berättelser.

[CIT0013] Jacelon C. S (1995). The effect of living in a nursing home on socialization in elderly people. Journal of Advanced Nursing.

[CIT0014] Kohler Riessman C. K, Quinney L (2005). Narrative in social work, a critical review. Qualitative Social Work.

[CIT0015] Kvale S (1997). Den kvalitativa forskningsintervjun.

[CIT0016] Labov W (1972). Language in the inner city.

[CIT0017] Linell P (1994). Transkription av tal och samtal: Teori och praktik. Arbetsrapport från tema K 1994:9.

[CIT0018] Linell P (2004). Essential of dialogism. Aspects and elements of a dialogical approach to language, communication and cognition.

[CIT0019] Linell P (2012). Samtalskulturer. - Kommunikativa verksamhetstyper i samhället.

[CIT0020] Mayeroff M (1999). On caring.

[CIT0021] McCance T. V, McKenna H. P, Boore J. R. P (2001). Exploring caring using narrative methodology: An analysis of the approach. Journal of Advanced Nursing.

[CIT0022] Mead G. H, Morris C. W (1967). Mind, self and society. From the standpoint of a social behaviorist.

[CIT0023] Meinow B, Parker M. G, Thorslund M (2011). Consumers of eldercare in Sweden: The semblance of choice. Social Science & Medicine.

[CIT0024] Merleau-Ponty M (1962). Kroppens fenomenologi.

[CIT0025] Mishler E. G (1984). The discourse of medicine: Dialectics of medical interviews.

[CIT0026] Nixon A, Power C (2007). Towards a framework for establishing rigor in a discourse analysis of midwifery professionalization. Nursing Inquiry.

[CIT0027] Polkinghorne D. E (1988). Narrative knowing and the human sciences.

[CIT0028] Porter E. J, Clinton J. F (1992). Adjusting to the nursing home. Western Journal of Nursing Research.

[CIT0029] Ricoeur P (1976). Interpretation theory: Discourse and surplus of meaning.

[CIT0030] Ricoeur P (1983). Temps et récit, I–III.

[CIT0031] Riessman C, Gubrium J. F, Holestein J (2002). Analysis of personal narratives. Handbook of interviews research. Context and method.

[CIT0044] Riessman C (2008). Narrative methods for the human sciences.

[CIT0032] Sarvimaki A (2006). Well-being as being well—a Heideggerian look at well-being. International Journal of Qualitative Studies on Health and Well-Being.

[CIT0036] SFS, (2003:460) The Swedish Act concerning ethical reveiw of research involving humans.

[CIT0033] Svensson A.-M, Bergh I, Jacobsson E (2011). Older Peoples’ descriptions of becoming and being respite care recipients. Journal of Housing For the Elderly.

[CIT0034] Svensson O, Hallberg L. R.-M (2011). Hunting for health, well-being, and quality of life. International Journal of Qualitative Studies on Health and Well-being.

[CIT0035] The National Board of Health and Welfare (2011). Vad tycker de äldre om äldreomsorgen? En rikstäckande undersökning av äldres uppfattning om kvaliteten i hemtjänst och äldreboenden 2011.

[CIT0037] Tornstam L, (Producer) (2007). This is an Internet publication. Please refer to the publication, with it's URL, as: Retrieved from Social Attitudes Toward Old-age Retirees in a 23-year Perspective.

[CIT0038] Tornstam L (2011). Ålrandets psykologi.

[CIT0039] Wånell S.-E, Thorslund M, Wånell S.-E (2008). Förebyggande insatser på äldre dar. Åldrandet och äldreomsorgen.

[CIT0040] Wiklund L (2003). Vårdvetenskap i klinisk praxis.

[CIT0041] Winter Jørgensen M, Phillips L (2011). Diskursanalys som teori och metod.

[CIT0042] WMA (Producer) (2008). World medical association declaration of Helsinki, ethical principles for medical research involving human subjects. http://www.wma.net/en/30publications/10policies/b3/17c.pdf.%20Retrieved%20from%20net/en/30publications/10policies/b3/17c.pdf.

[CIT0043] World Health Organisation (1946). Constitution of the World Health Organisation.

